# Supplementary material for: Protocol for a QUALICOPC study in a Malaysian setting: Primary care pharmacy services system performance and T2DM older adults’ quality of life evaluation
Source: PLoS One. 2025 Jun 12;20(6):e0323183. doi: 10.1371/journal.pone.0323183 (PMC12161544; doi:10.1371/journal.pone.0323183)
Supplement: S1 Table — (DOC) [file pone.0323183.s001.doc]

| **Where** | **What** | **How** | **Which** | **Why** |
| --- | --- | --- | --- | --- |
| Primary care pharmacy system (structure) level | (1) Planning  (Consider flexibility level of pharmacy organization) | Steady state | A pharmacy does not offer services beyond traditional dispensing activities; suggests offering immunization services | - To evaluate whether a value-added pharmacist service should pursue or not |
|  |  | Operational | A pharmacy is beginning to expand offerings to increase the number of customers but not providing a value-based services |  |
|  |  | Structural | A pharmacy that has developed at least one-value-based service and begun to implement internal infrastructure to support the service |  |
|  |  | Strategic | A pharmacy has successfully implemented value-based services and has infrastructure and processes in place for sustainability; suggests partnering with providers for diabetes management services |  |
|  | (2) Outcome Evaluation  (Important to create an evaluation plan early to have the necessary infrastructure in place for successful outcome reporting) | Process | Examples: Number and type of medication-related problems identified and resolved | - To determine the effectiveness and value of services (a good practice to obtain feedback from key stakeholders about outcomes)  -> Combination of Process + Clinical + Economic Outcomes (To be able to have a full picture of the impact of a service)  -> When Clinical + Humanistic Outcomes are achieved, Financial Outcomes are in lockstep with them |
|  |  | Clinical | Examples: Systolic and diastolic blood pressure, HbA1c and lipid levels |  |
|  |  | Economic/ Financial | Examples: Total health care costs of medications, hospitalizations or emergency department visits |  |
|  |  | Humanistic | Examples: Patient satisfaction |  |
|  | (3) Monitoring and Sustaining  (Pharmacy organizations will advance the profession and best serve the community when it develops sustainable services that can treat and monitor patient progress for positive outcomes for a long term) | Implementation Science | - Useful approach: Consolidated Framework for Implementation Research (CFIR) model  - Examples of studies: (i) Positive impact on pharmacy and patients when Appointment Based Model (ABM) designed using CFIR approach, (ii) clinically proven asthma service in Australian community pharmacy, (iii) breastfeeding-related health services in community pharmacies, (iv) mental health medication management in Australian community pharmacies | - To ensure service longevity (evidence strength and quality advantage; adaptability; trialability; design quality and packaging; and cost)  - Have demonstrated success in service design and monitoring |

**S1 Table. Steps for successful implementation of value-added pharmacist services at the primary care pharmacy system (structure) level^a,b^.**

^a^Adapted from Desselle et. al. (2019).

^b^Based on 5W1H framework; need to be done whenever appropriate.
