# Supplementary material for: Protocol for a QUALICOPC study in a Malaysian setting: Primary care pharmacy services system performance and T2DM older adults’ quality of life evaluation
Source: PLoS One. 2025 Jun 12;20(6):e0323183. doi: 10.1371/journal.pone.0323183 (PMC12161544; doi:10.1371/journal.pone.0323183)
Supplement: Attachment — Submitted filename: Response_to_Reviewers_auresp_2.docx [file pone.0323183.s005.docx]

Reviewers' comments:

Reviewer's Responses to Questions

Comments to the Author

1. Does the manuscript provide a valid rationale for the proposed study, with clearly identified and justified research questions?

The research question outlined is expected to address a valid academic problem or topic and contribute to the base of knowledge in the field.

Reviewer #1: No

Reviewer #2: Yes

2. Is the protocol technically sound and planned in a manner that will lead to a meaningful outcome and allow testing the stated hypotheses?

The manuscript should describe the methods in sufficient detail to prevent undisclosed flexibility in the experimental procedure or analysis pipeline, including sufficient outcome-neutral conditions (e.g. necessary controls, absence of floor or ceiling effects) to test the proposed hypotheses and a statistical power analysis where applicable. As there may be aspects of the methodology and analysis which can only be refined once the work is undertaken, authors should outline potential assumptions and explicitly describe what aspects of the proposed analyses, if any, are exploratory.

Reviewer #1: No

Reviewer #2: Yes

3. Is the methodology feasible and described in sufficient detail to allow the work to be replicable?

Descriptions of methods and materials in the protocol should be reported in sufficient detail for another researcher to reproduce all experiments and analyses. The protocol should describe the appropriate controls, sample size calculations, and replication needed to ensure that the data are robust and reproducible.

Reviewer #1: No

Reviewer #2: Yes

4. Have the authors described where all data underlying the findings will be made available when the study is complete?

The PLOS Data policy requires authors to make all data underlying the findings described in their manuscript fully available without restriction, with rare exception, at the time of publication. The data should be provided as part of the manuscript or its supporting information, or deposited to a public repository. For example, in addition to summary statistics, the data points behind means, medians and variance measures should be available. If there are restrictions on publicly sharing data—e.g. participant privacy or use of data from a third party—those must be specified.

Reviewer #1: Yes

Reviewer #2: Yes

5. Is the manuscript presented in an intelligible fashion and written in standard English?

PLOS ONE does not copyedit accepted manuscripts, so the language in submitted articles must be clear, correct, and unambiguous. Any typographical or grammatical errors should be corrected at revision, so please note any specific errors here.

Reviewer #1: No

Reviewer #2: Yes

6. Review Comments to the Author

Please use the space provided to explain your answers to the questions above and, if applicable, provide comments about issues authors must address before this protocol can be accepted for publication. You may also include additional comments for the author, including concerns about research or publication ethics.

You may also provide optional suggestions and comments to authors that they might find helpful in planning their study.

(Please upload your review as an attachment if it exceeds 20,000 characters)

Reviewer #1: The article is confusing, and its objectives, methods, and what will be conducted are not clearly stated. It needs to be thoroughly revised or rewritten.

*Author`s feedback:*

Thank you for your comments.

The objectives are added to the protocol as below:

‘This paper sought to conduct a more in-depth exploration of the quality of public PC pharmacy services in Malaysia as assessed by the experience of T2DM older adults or their caregivers and pharmacy staff, besides exploring some aspects of healthcare delivery that clients (could be either patients or their caregivers) value. Comparisons are also will be made between two types of major services provided by the public PC pharmacy (i.e. pharmaceutical care a.k.a. DMTAC and pharmacy VAS) as we hypothesize experiences will differ across these patient sub-groups. The secondary objective includes assessing the QOL of older adults with T2DM who are the respondents in this study and receiving the main pharmacy services in Malaysia's public PC pharmacy system (pharmaceutical care or pharmacy VAS) which is potentially useful in both the clinical encounter and quality improvement. Finally, this paper will provide possible solutions to inform healthcare reform activities moving forward‘.

These sentences also are added to the method section for clarity:

`The author highlights the lack of patient HRQOL and economic benefits evaluation of pharmacists’ roles in providing clinical services for diabetes care in a Malaysian public PC pharmacy. Furthermore, the author addressed that public PC pharmacies in a Malaysian setting are in the strategic planning in which a pharmacy has successfully implemented value-based services and has infrastructure and processes in place for sustainability, thus suggesting partnering with providers for diabetes management services. This strategy may be evaluated by clinical and humanistic outcomes [20]. Desselle et. al. (2019) emphasized that when clinical and humanistic outcomes are achieved, financial outcomes also align with them [20]. Thus, the author uses the QOL questionnaire to measure clinical outcomes and QUALICOPC questionnaires to measure human satisfaction or humanistic outcomes‘.

I do not clearly understand how patient quality of life, as assessed by the EQ-5D scale, can be affected by the effectiveness of primary care (PC) pharmacies. It would be necessary to collect numerous other variables, such as patient comorbidities, which have a major impact on quality of life, to study such an association after adjustment. Furthermore, quality of life assessment is a secondary objective and not the main focus of the study. The title is unclear and should be revised, for instance: Primary Care Pharmacy Services System Performance for T2DM Older Adults: Protocol for a Cross-Sectional Study.

*Author`s feedback:*

Thank you for your suggestions.

I added these sentences to the protocol:

`In a Malaysian public PC pharmacy setting, the main services offered for older adult diabetes patients include pharmaceutical care and Pharmacy Value Added Services (VAS). The offered pharmaceutical care is recognised as Diabetes Medication Therapy Adherence Clinics (DMTAC). Meanwhile, some examples of Pharmacy VAS include Drive-through pharmacy, Medicine by Post, SMS Take&Go, Appointment Card, and medicine lockers, commonly referred to as Medibox [21]‘.

`The author highlights the lack of patient HRQOL and economic benefits evaluation of pharmacists' roles in providing clinical services for diabetes care in a Malaysian public PC pharmacy. Furthermore, the author addressed that public PC pharmacies in a Malaysian setting are in the strategic planning in which a pharmacy has successfully implemented value-based services and has infrastructure and processes in place for sustainability, thus suggesting partnering with providers for diabetes management services. This strategy may be evaluated by clinical and humanistic outcomes [20]. Desselle et. al. (2019) emphasized that when clinical and humanistic outcomes are achieved, financial outcomes also align with them [20]. Thus, the author uses the QOL questionnaire to measure clinical outcomes and QUALICOPC questionnaires to measure human satisfaction or humanistic outcomes ‘.

*Additional explanation from the author:*

The EQ-5D-5L questionnaire is suitable for use in clinical environments. Higginson et al. (2001) highlighted the significance of quality of life (QOL) measures in clinical practice. QOL measures help transition the focus from the disease to the patient. These measures are useful during clinical interactions and for quality improvement efforts. Marten et al. (2022) reviewed literature showing that the EQ-5D-3L remains feasible and applicable for older individuals.

Comorbidities of patients will not be included in the data collection. Since the data will be collected specifically from the pharmacy department, the focus will be solely on the types of medications patients receive. Medication data will be incorporated into questionnaires.

The study is a QUALICOPC study of a PC pharmacy services system performance, thus the QUALICOPC maintains in the title because it adapted QUALICOPC questionnaires previously used in over 30 countries. The study uses the mechanism of inter-relations between different levels and features, which is referenced from Schäfer et al. (2011), a QUALICOPC survey. The questionnaires will be validated for the Malaysian context. The study focuses on evaluating the quality of life (QOL) of older adults, which may be beneficial for the quality of pharmacy services improvement. Thus, the title should reflect the emphasis on QOL.

Table 1

Are the various types of services listed in Table 1 currently implemented by pharmacies in Malaysia? If not, the authors should specify this.

*Author`s feedback:*

Yes, some types of services listed in Table 1 are currently implemented by pharmacies in Malaysia. Thank you for your suggestion.

I added these sentences in the protocol:

In a Malaysian public PC pharmacy setting, the main services offered for older adult diabetes patients include pharmaceutical care and Pharmacy Value Added Services (VAS). The offered pharmaceutical care is recognised as Diabetes Medication Therapy Adherence Clinics (DMTAC). Meanwhile, some examples of Pharmacy VAS include Drive-through pharmacy, Medicine by Post, SMS Take&Go, Appointment Card, and medicine locker, commonly referred to as Medibox [21].

Sample size: The calculation provided (which cannot be verified due to the lack of details regarding the desired precision in estimating diabetes prevalence) is irrelevant to this study. This is not a prevalence study. There is no need for a sample size calculation unless there is a specific hypothesis related to the primary endpoint.

*Author`s feedback:*

Thank you for your suggestion. The protocol outlines that hypothesis testing will focus on the primary endpoint involving QUALICOPC survey data. The survey collects responses from both clients and staff. Analysis of factors associated with comparison groups will utilize simple and multiple logistic regression. Thus, it is rational for the sample size of the client responses should be calculated.

I revised the protocol:

`The determination of the sample size for the QUALICOPC study on clients (T2DM older adults or their caregivers) using the same formula as a cohort study, by comparing two prevalence rates, as the data was collected through an analytical cross-sectional study using questionnaires [25]. An online calculator which can be found at https://riskcalc.org/samplesize/ is used to estimate the sample size [25], with the Malaysian population's prevalence of older adults with T2DM being 33.45% being the focus [26]. This online calculator uses the formula provided by Fleiss et. al. (1980) [27] for the calculation of sample size [25]. This study utilized published data on the prevalence of older adults in Malaysia for sample size calculation due to the lack of information on the proportion or prevalence of the specific comparison groups. To ensure accuracy, the sample size was calculated to be 39 in each group (2-sided significance level set at 0.05, power of 0.8, and the sample size ratio is 1). In this study, we have three (3) different groups which are `DMTAC`, `pharmacy VAS`, and `not enrolled with either DMTAC or pharmacy VAS`. Thus, the total sample size should be 117. With a 10% increase to account for potential dropouts, non-responses, and missing records, a recommended total sample size of 144 (each group has 48 samples) is sufficient for evaluating the performance in terms of client experience, and 48 samples (each group has 16 samples), is sufficient for evaluating the performance in terms of client value of the current public PC pharmacy services system. The calculation of each group has 16 samples is by the assumption that the sample proportion distribution approximates a normal distribution with the formula of np≥5 and n(1-p)≥5, in which `p` is a fixed value of the population proportion and `n` is the size of the random sample [28]`.

The calculation from the online calculator is as follows:

Methods: The article would benefit from greater clarity regarding the study hypothesis, the primary objective of the study (with the associated endpoint), and the secondary objectives (with their corresponding endpoints).

*Author`s feedback:*

Yes. That is true.

The hypothesis was already in the protocol as below:

‘Data gathered from the system (structure), service provision, and user levels can provide valuable insights into the strengths and areas for improvement of pharmacy services, specifically DMTAC and pharmacy VAS, within a public PC pharmacy system in Malaysia. Fig 1 explains the hypotheses for both clients and pharmacy staff. The below sentences are added to the protocol:

`The outcome from QUALICOPC questionnaires for clients and the health-related quality of life (HRQOL) questionnaire is dependent on the DMTAC enrollment status group and Pharmacy VAS enrollment status group (Fig 1). Outcomes from the QUALICOPC questionnaire for staff are dependent on the pharmacy staff occupation group, while perception of staff work satisfaction is dependent on staff workload (Fig 1)`.

The objectives are added to the protocol as below:

‘This paper sought to conduct a more in-depth exploration of the quality of public PC pharmacy services in Malaysia as assessed by the experience of T2DM older adults or their caregivers and pharmacy staff, besides exploring some aspects of healthcare delivery that clients (could be either patients or their caregivers) value. Comparisons are also will be made between two types of major services provided by the public PC pharmacy (i.e. pharmaceutical care a.k.a. DMTAC and pharmacy VAS) as we hypothesize experiences will differ across these patient sub-groups. The secondary objective includes assessing the QOL of older adults with T2DM who are the respondents in this study and receiving the main pharmacy services in Malaysia's public PC pharmacy system (pharmaceutical care or pharmacy VAS) which is potentially useful in both the clinical encounter and quality improvement. Finally, this paper will provide possible solutions to inform healthcare reform activities moving forward‘.

Inclusion criteria: Why do the authors include the criterion "patients and/or have a spouse involved"? Similarly, participation in another study should not be considered an exclusion criterion.

*Author`s feedback:*

Thank you for your questions. Most older adults with Type 2 Diabetes Mellitus (T2DM) in Malaysia visit public pharmacies accompanied by a spouse or caregiver, especially if they use a wheelchair. These patients often rely on caregivers for follow-up medication collection, while they may remain at home. The study focuses specifically on the follow-up aspect of medication collection. Updated information has been incorporated into the inclusion criteria for the study.

`Clients which include patients aged 60 years or older who have been diagnosed with T2DM and/or are currently taking metformin as part of their treatment, have previously received medications during a DMTAC or Pharmacy VAS appointment (if enrolled in DMTAC or Pharmacy VAS only), have given informed consent and who can understand English and/or Malay, have an experience of at least once took medication from the public PC pharmacy as well as have expressed a desire to participate will be eligible to participate in this study. The adult caregivers aged 18 years and above of this patient criteria are also considered clients. Patients who are under the age of 60, have been diagnosed with Type 1 diabetes, are currently using acute medications, are enrolled in DMTAC and pharmacy VAS at the same time, or are participating in other research studies will not be eligible to participate in this study. The caregiver with this patient criteria will be excluded from this study`.

*Additional explanation from the author:*

To avoid confusion and misleading or contamination of the results, participation in any other study will be excluded.

What is the difference between the experience survey and the values survey?

*Author`s feedback:*

Thank you for your question. Client Experience is related to the Primary Care Pharmacy Domain, in which clients rated either `yes` or `no` based on their experience. In contrast, Client Value is related to Quality Domain, in which clients rated either `very important`, `important`, `not important`, or `somewhat important`. Both questionnaires are concerning the assessment of 5 factors (i) accessibility, (ii) continuity and coordination, (iii) communication and patient-centered care, (iv) patient activation, and (v) comprehensiveness. The respondents for client questionnaires are either T2DM older adults or their caregivers.

The questionnaires for patients and pharmacists should be better described in a dedicated section or paragraph, including the number of questions, the different items evaluated, etc.

*Author`s feedback:*

Thank you for your question. The staff questionnaire will be based on two published papers. The specific number of questions is not yet determined. Finalisation of the questionnaire will occur after validation is completed.

Reviewer #2: No concern on research or publication ethics

*Author`s feedback:*

Thank you for your comment. It is mentioned in the ‘Means to Disclose Information on Research‘.

Just wondering if the staff satisfaction survey includes workplace culture/environment

*Author`s feedback:*

Thank you for your suggestion. The staff questionnaire will be based on two published papers (References 23 and 30). These references contain only 6 questions focused on work satisfaction perception. The questionnaire has been validated internationally. Adaptations will be made to tailor the questionnaire to a Malaysian context.

Moving forward, can consider covering a wider range of populations, for example, all elderly (GMTAC) as the prevalence of elderly in Malaysia is more prevalent than DM in the elderly. Malaysia is moving toward an aged nation soon.

*Author`s feedback:*

Thank you for your suggestion. This study assesses pharmacy services for older adult diabetes patients in Malaysia, including those with hyperlipidemia and hypertension. It aims to identify the strengths and weaknesses of these services specifically for this demographic. The focus on older adults with diabetes is crucial due to the rising prevalence of the disease in Malaysia. The increasing rates of diabetes among older adults have significant economic and health implications. This issue is not encountered in Malaysia; it is a global concern.

7. PLOS authors have the option to publish the peer-review history of their article (what does this mean?). If published, this will include your full peer review and any attached files.

If you choose “no”, your identity will remain anonymous but your review may still be made public.

Note: Highlighted in yellow are the changes that have been made in the protocol.
